# Population and Colony-Level Determinants of Tertiary Sex Ratio in the Declining Barn Swallow

**DOI:** 10.1371/journal.pone.0056493

**Published:** 2013-02-13

**Authors:** Nicola Saino, Maria Romano, Diego Rubolini, Manuela Caprioli, Roberto Ambrosini, Giuseppe Boncoraglio, Luca Canova

**Affiliations:** 1 Dipartimento di Bioscienze, Università degli Studi di Milano, Milano, Italy; 2 Dipartimento di Biotecnologie e Bioscienze, Università di Milano-Bicocca, Milano, Italy; 3 Dipartimento di Biologia e Biotecnologie, Università degli Studi di Pavia, Pavia, Italy; University of Tasmania, Australia

## Abstract

Sex ratio of adults (tertiary sex ratio, TSR) is a major feature of animal populations with consequences for their behaviour, genetic structure and viability. Spatial and temporal variation in TSR occurs within species but the mechanisms behind it are poorly understood. In this long-term study of a declining population of a socially monogamous, colonial, migratory bird, the barn swallow (*Hirundo rustica*), we first analyzed population-level variation in TSR ( = proportion of males) of yearlings at sexual maturation in relation to ecological conditions as gauged by annual survival rate of adults. TSR was male-biased both among yearlings and older individuals, but male bias of yearlings was more pronounced after years with larger decline in adult survival. Thus, male offspring were less susceptible to the adverse ecological conditions that cause increased mortality. Dispersal and settling site decisions can have major consequences on fitness via the effects of local TSR on mating and sperm competition. Breeding barn swallows are highly philopatric while natal dispersal is high and, together with mortality, is the main determinant of colony TSR. We thus also investigated the mechanisms of breeding colony choice by yearlings and found that TSR of new-settlers in a given colony and year was negatively predicted by TSR of returning, early arriving older individuals in that year, but not by overall TSR at the colony in the previous year. This suggests that in our male-biased population new-settler males respond to local TSR upon arrival to choose the sites with larger breeding opportunities. Hence, variation in ecological conditions as reflected by adult survival can shift the TSR of individuals recruiting into a local population, with potentially various demographic consequences. However, breeding site choice based on TSR tends to homogenize TSR at a population level likely by facilitating settling of dispersing males in colonies with less male-biased TSR.

## Introduction

Sex ratio of reproductively mature individuals (tertiary sex ratio, TSR) dictates major features of populations of organisms, from mating systems and sexual selection regimes, to genetically effective size and growth rates, and can thus ultimately affect population trends and viability [1]–[4]. Ever since the insights of C. Darwin [5] and R. Fisher [6] it has been held that frequency-dependent fitness advantages of producing offspring of the rarer sex should stabilize population sex ratios close to parity, and this is expected to occur independently of the individual allocation strategies to either sex, which may vary among breeding adults [7], [8]. However, studies of TSR have repeatedly disclosed deviations from parity and that general patterns of variation in such deviations exist among species or higher order taxa (e.g. vertebrate classes). For example, in birds TSR is frequently male-biased, though opposite female-biases are not exceptional, whereas female-bias prevails in mammals [3], [9]. On the other hand, bias in TSR may also vary among closely related taxa or even within species [10], possibly because the impact of several, potentially contrasting mechanisms that affect TSR depends on environmental conditions [11], [12]. Interestingly, male-bias has been shown to increase in association with negative trends particularly in small, isolated bird populations [3], [13], although the flow of causation, i.e. whether variation in TSR is a cause or, conversely, a consequence of population decline, is ancipitous and its direction remains to be elucidated.

In general, the mechanisms that generate deviations of TSR from parity are very poorly understood. In birds, population-level biased primary (at fertilization) or secondary (hatching/fledging) sex ratios are relatively uncommon [3], and male-biased TSR is thus thought to result mainly from larger female mortality due to larger investment in reproduction [14], although it may also occur before sexual maturation [15]. Sex-related mortality is indeed to be expected owing to extensive genetic, physiological and ecological differences between the sexes [3], [16], [17]. The heterogametic sex (females in birds) may incur the cost of the expression of deleterious recessives on the unguarded sex chromosome. Males and females may experience different costs of socio-sexual activities and reproduction, be more exposed to predation or inherently more susceptible to parasitism or, due to social dominance, differ in access to limiting food. Sex-biased dispersal, whereby female birds are more likely to disperse and do so over larger distances than males, is an additional source of differential mortality because dispersal entails viability penalties [2], [18].

Changing selection regimes can thus cause TSR to vary in time and this is particularly true because skews in TSR go uncompensated, as parents typically do not adjust their sex allocation to current population TSR [19], [20]. However, whether an increase or a reduction in the relative abundance of males under changing ecological conditions should be expected may be difficult to predict owing to the complexity of the mechanisms controlling sex-related mortality rates and recruitment of dispersing individuals.

In fact, dispersal is a major ultimate factor affecting local genetic and demographic processes [21]–[24] and most often involves young individuals [25]. In socially monogamous systems like the vast majority of bird species, dispersal is expected to tend to homogenize TSR among e.g. local demes in habitat fragments or breeding colonies. This is the case because TSR can affect mating opportunities and sperm competition: individuals will tend to settle where same-sex competitors for a given number of potential mates are relatively less frequent [4], although alternative scenarios are plausible when, for example, high quality male “hotshots” promote the aggregation of other males [26]. The direct and kin selection effects of such dispersal and settling decisions based on TSR, however, may be asymmetrically distributed between the sexes. In male-biased bird populations, dispersing males should accrue marked benefits from settling where TSR is closer to parity, because they will experience lower mating and sperm competition, as well as post-fertilization sexual selection mediated by infanticide. On the other hand, the payoff of deciding where to settle based on local TSR in a male-biased population is less straightforward for females. While male-biased TSR will offer females with more opportunities for social and extra-bond mate choice, it will also entail costs via social aggression, sexual harassment and infanticide. Most importantly, however, in a male-biased population availability of social mates will be limiting to males but not to females, leading to expect smaller female choosiness of breeding site based on local TSR.

Variation in TSR is thus a key determinant of the evolution and dynamics of populations, and thus of their viability. However, our knowledge of the factors that determine temporal and spatial variation of TSR both at the population and at the local levels is still very sparse. This is particularly true for birds, partly because of the notorious difficulties inherent in studies of vagile organisms with large natal and sometimes also breeding dispersal [3], [27], [28].

In this 16-years study of a declining population [29]–[31] of the socially monogamous, colonial, long-distance migratory barn swallow (*Hirundo rustica*) we first analyze how TSR changes according to age, by comparing 1-year old individuals (‘yearlings’) at sexual maturation to older individuals. Second, we test whether TSR of yearlings at the time of recruitment depends on harshness of ecological conditions experienced during their first year of life. As a reliable proxy for severity of extrinsic conditions we used survival rate of the adults. Secondary sex ratio (at fledging) is close to parity in our study population, though with annual fluctuations [20], but we found it to be significantly male-biased already among yearlings, suggesting larger female mortality before sexual maturation. Therefore, we expected TSR of yearlings to be more male-biased after years with larger adult mortality because ‘baseline’ female-biased mortality during the first year of life should be exacerbated under adverse conditions. Finally, we investigate the mechanisms mediated by local recruitment that determine colony TSR. In the barn swallow, the frequency of natal dispersal (i.e. breeding in a colony different from the original one) is very high, with only 5–10% of the offspring returning to their original colony [32]–[34]. Conversely, breeding dispersal is very low (i.e. breeders do not move to a different colony in consecutive years). Hence, colony-level variation in TSR is mainly due to recruitment of yearlings, mostly originating from other colonies, and mortality of local breeders. Temporal segregation in arrival dates occurs between philopatric breeders and yearling recruits, with the latter arriving on average few weeks later than the former [32], [35]. Thus, when yearlings reach their breeding area, older individuals have already arrived and may provide a cue to local TSR that yearlings can use upon arrival to optimize breeding colony choice (‘arrival TSR’ hypothesis). Alternatively, offspring may choose their breeding site already in their spring/summer of birth, before undertaking their first migration (‘departure TSR’ hypothesis). This could allow to avoid having to spend energy and time to choose the breeding site upon return from migration, in the haste of their first breeding season. These hypotheses were contrasted by testing whether TSR of yearlings in any particular colony was better negatively predicted 1) by overall (yearlings+older individuals) TSR in the previous year (departure TSR hypothesis), or 2) by TSR of older, early arriving individuals in the year of settlement (arrival TSR hypothesis).

## Methods

This study is based on data on adult barn swallows collected in 16 years over the period 1993–2010 at 14 colonies ( = farms) near Milano (N Italy). The duration of time series at individual colonies ranged between 4 and 9 years (see Results for sample sizes). The 14 study farms were scattered over a ca. 250 km^2^ (minimum convex polygon) area located in the central part of the Po valley, a large (>40.000 km^2^), intensively cultivated, homogeneous lowland region in northern Italy. The number of farms with breeding barn swallows that existed within the ca. 250 km^2^ area where our study colonies were located can be estimated to exceed 150 (based on density of farms with colonies reported in [36]), while the number of breeding pairs in the Po valley can be estimated to be in the order of hundreds of thousands (based on calculations for the Regione Lombardia in [30]). Thus, our study colonies were scattered among several other colonies, and our study area was located in a central position in the Po valley, which hosts a very large (though rapidly declining) breeding barn swallow population. Mean natal dispersal distances of barn swallows are in the order of several kilometers, hugely vary among individuals (ranging between 0 and hundreds of kilometers), and have been estimated to be twice as large for females compared to males ([33]; our unpublished data). For these reasons, our study colonies did not represent a small, isolated or marginal population and were rather representative of the large barn swallow population that breeds in the Po valley.

Barn swallows in northern Italy have suffered a sharp population decline (4–9% per year) during the last decades [29], [31].

At each study colony we did repeated capture sessions to individually mark the adults throughout spring (April–June) in all study years. Barn swallows usually spend the night inside the rural buildings where they breed, and we could therefore efficiently capture all the individuals of the colony by putting up nets at all exits before dawn. Sex was identified according to a number of cues including presence of a cloacal protuberance (males) or an incubation patch (females), length of the outermost tail feathers, and observation of parental (incubation) and socio-sexual behaviour (song) at the nest, and confirmed at upon repeated captures [32]. In few cases of uncertain assignment, sex was identified by molecular techniques [20].

Barn swallows in our as well as in neighbouring populations in northern Italy and southern Switzerland, and in other European regions show very high breeding philopatry, while natal philopatry is very low [32], [33], [37], [38]. High breeding philopatry and capture probability of all individuals of the colony (see above) imply that we could assume that in any given year unmarked birds (i.e. birds that had not been captured in previous years) were 1-year old adults (i.e. individuals hatched in the previous spring-summer; hereafter ‘yearlings’). Conversely, all the individuals that had already been captured in previous year(s) were two- or more years old (hereafter ‘older individuals’) (see [37], [38] for further details). This procedure implies that in the first study year of any individual colony, sex but not age of the birds was known. Thus, sex ratios (independent of age) were available for 81 year×colony events while age-class specific TSR and population trends were available for 67 years×colonies events.

### Statistical analyses

Adult survival was expressed as log_e_ (N*_t+1_*/N*_t_*), where N*_t+1_* is the number of individuals (males+females) that were already present as adults in year *t* and survived till year *t*+1, and N_t_ is the number of adults in year *t*. The log-transformed value of N*_t+1_*/N*_t_* ( = λ_t_) was used in the analyses in order to avoid any problems arising because of serial correlation between the λ_t_ values [39]. However, analyses run using untransformed λ_t_ values led to strictly similar results. As a proxy for ecological conditions that potentially affected TSR of yearlings in any year *t* we used survival rates of adults from year *t*-1 till year *t*. This likely represents a good proxy of ecological conditions experienced by offspring during their first year of life because adults and offspring share the same wintering grounds and likely also migration routes, though with some asynchrony in timing of migration [33]. In fact, we considered that this is a much better proxy for conditions experienced year-round than meteorological (e.g. rainfall) or bioclimatic (e.g. NDVI) [35] indicators because of the uncertainty on the relevant areas passed during migration or wintering and time periods. Colony growth rate was expressed as log_e_ (C*_t+1_*/C*_t_*), where C*_t+1_* is the number of adults in the colony in year *t*+1 and C_t_ is the number of adults in the previous year. Adult mortality during the breeding season is very low (our unpublished data) and we are thus confident that growth rates and TSR computed using the total of individuals that were captured during the whole breeding season were accurately estimated. We could not analyze population TSR (yearlings+older individuals) in relation to population growth rate because these variables are inherently correlated. The relationship between yearling TSR and adult survival was analyzed over the 8 years when sample size of yearlings exceeded 180, as in the other years the available sample size was much smaller (40–100) and TSR estimates could therefore be affected by random noise. No such precaution was needed in colony-level analyses because colony-level TSR was known with very small error, given that colonies were sampled exhaustively.

Sex ratios were analyzed in generalized (GLM) or generalized linear mixed (GLMM) models with a binomial error distribution and a logit link-function. In mixed models, colony and/or year were included as random factors when appropriate (see Results). Time could not be included as predictor in the analyses at population level because of collinearity with survival of adults. Statistical analyses were run with SPSS 13.0 and SAS 9.2 statistical packages.

### Ethics statement

Upon capture, barn swallows were kept in cloth bags in a safe position not accessible to predators, as is standard practice in bird ringing studies. All individuals were sexed and released as soon as possible, usually within 1 hour of capture. After being released, swallows behaved normally and observations at the nest on hundreds of individuals confirmed that they resumed their normal breeding activities. The study was carried out under ringing permit 0665 released by the Istituto Nazionale per la Fauna Selvatica.

## Results

### Population-level analyses

Males significantly outnumbered females (total number of annual male records = 2877, female records = 2446; TSR = 0.540; null binomial GLMM with farm and year as random factors: t_13_ = 7.67, P<0.0001). Age-specific TSR could be analyzed over 13 years in the 14 colonies (duration of time series: 3–8 yrs). An excess of males existed already among yearlings (total records = 2572; TSR = 0.534; t_13_ = 3.33, P = 0.0054) and persisted among older individuals (total records = 1707; TSR = 0.547; t_13_ = 4.98, P = 0.0003; GLMM as above). No significant variation in TSR between yearlings and older individuals emerged (GLMM as above: effect of age: F_1,107_ = 0.69, P = 0.409).

**Figure 1:**
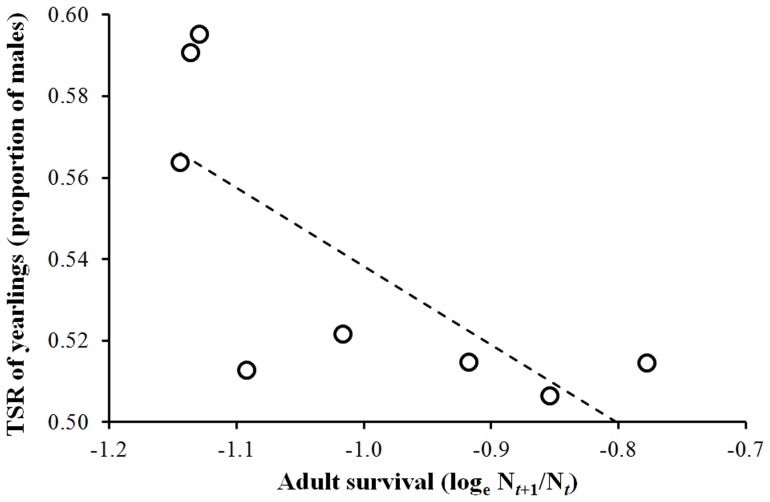
TSR of yearlings in the population in any (*t*+1)-th study year in relation to adult survival between year *t* and year *t*+1. The analysis was restricted to the years when more than 180 yearlings were sampled (see *Statistical analyses*).

Population-level TSR of yearlings in year *t+1* in relation to adult survival between year *t* and *t*+1 was analyzed over 8 years. Mean survival recorded in these years was 0.371 (0.023) for males and 0.365 (0.018) for females. The mean of annual yearling TSR in these years was 0.540 but varied considerably among years (range: 0.506–0.595; interquartile range: 0.071; see Fig. 1). As expected, a clear negative relationship between yearling TSR and adult survival emerged (GLM: χ^2^_1_ = 4.95, P = 0.026, coefficient = −0.707 (0.318); Fig. 1). An inspection to Figure 1 shows, however, that the effect of survival was non-linear, with a discontinuity occurring for growth rates around −1.1 (i.e. for survival = 0.33): TSR was significantly more male-biased below than above this threshold (GLM: χ^2^_1_ = 9.13, P = 0.0025).

### Colony-level analyses

In the overall sample of 14 colonies over the 16 study years, TSR was found not to vary among colonies (GLMM with year as random factor; effect of farm: F_13,53_ = 0.48, P = 0.926). Moreover, colony TSR was not predicted by the number of individuals in the colony (GLMM with colony and year as random factors: F_1,66_ = 0.23, P = 0.630).

**Figure 2:**
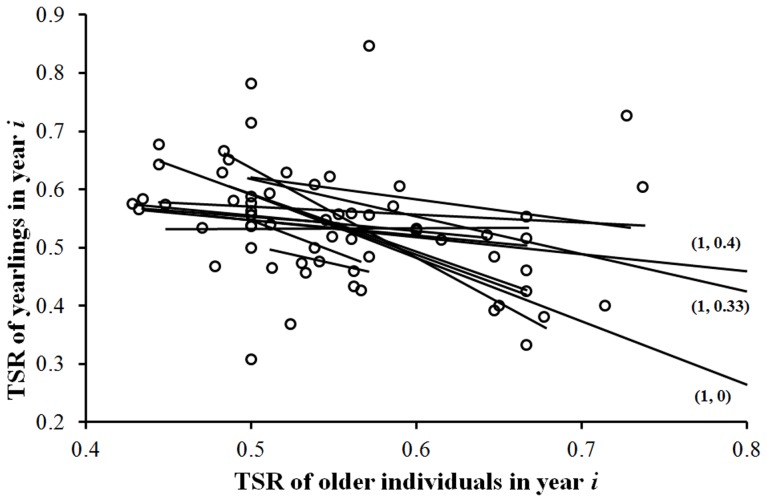
Yearlings TSR in relation to older individuals TSR recorded in the same year in 67 years×colony events. Continuous lines are least squares regressions for the 14 colonies included in the sample. Three data points with sex ratio of older individuals = 1 are not represented to better visualize the other data points. The coordinates (*x*, *y*) of those points are in parentheses.

Estimates of TSR of yearlings and older individuals were available for 67 colony×year events (range of years per colony: 3–8; number of annual records: 2306 males, 1973 females). There was a strong negative covariation between TSR of yearlings in a given colony and year and TSR of older individuals in the same year and colony (F_1,40_ = 14.98, P = 0.0004, coefficient = −1.73 (0.45); Fig. 2). Inclusion of colony growth rate as a covariate in this model did not reveal any significant effect (F_1,39_ = 1.92, P = 0.173) and left the negative effect of TSR of older individuals qualitatively unaltered (F_1,39_ = 16.261, P = 0.0002). In a different model, TSR of yearlings in a given year was not predicted by colony TSR (yearlings+older individuals) in the previous year (GLMM: F_1,40_ = 1.81, P = 0.186).

## Discussion

The first main novel findings of this study of a markedly declining population [29], [31] of barn swallows were that TSR was consistently male-biased among yearlings as well as older individuals, did not change between age classes and was homogeneous among colonies. Second, temporal variation in TSR of yearlings at the time of their recruitment as sexually mature individuals was negatively related to annual survival of adults, which we considered as a proxy for ecological conditions experienced by our population during the annual cycle. Third, TSR of yearlings that were newly recruited into any breeding colony was negatively predicted by contemporary TSR of older individuals that had already settled in that colony, but was not predicted by TSR of individuals breeding in that colony in the previous year.

The TSR of barn swallows measured on the breeding grounds (this study; [32]) and population sex ratio during wintering (including both young from the previous spring and adults; [40]) is male-biased, while sex ratio of fledglings does not significantly deviate from parity in most years, although it can episodically spike to male-biased. The mean of the annual nestling sex ratio is 0.518 (computed based on [20]) while the corresponding figure for yearlings recorded here was 0.540. TSR did not significantly change between yearlings and older individuals. These results suggest that male-bias in TSR arises at some stage during the first year of life. This interpretation is supported by the non-significant difference in life expectancy between the sexes after sexual maturation [34]. Present findings are thus consistent with the general pattern of a male-bias in TSR of birds. In addition, they provide evidence that male-bias does not primarily result from sex-related costs of reproduction, given that it arises before sexual maturation [15]. An alternative interpretation of the larger male-bias among sexually mature individuals compared to nestlings is female-biased dispersal, whereby the sex ratio of the offspring that dispersed from our study colonies was less male-biased compared to that of the yearlings that immigrated and were eventually recruited as breeding adults. This could occur, for example, if our study colonies belonged to a small, isolated population at the edge of the breeding range of barn swallows. Natal dispersal distances of barn swallow are larger for females than males ([33], [34]; our unpublished data) and females could therefore be more likely to emigrate from an isolated population. However, our study colonies were in fact scattered among several other colonies, and were located in the central part of an ecologically homogeneous farmland which hosts a very large (>100.000 breeding pairs) population (see Methods; see also [30]). Given the natal dispersal distance of barn swallows, the location of our study colonies, and the density of colonies (0.7 colonies km^−2^; [36]) in the ecologically homogeneous area where the study was conducted, it seems that our study colonies did not experience any isolation-by-distance or by ecological barriers. Thus, while sex-biased dispersal and isolation can obviously affect local TSR, we are convinced that these effects are not sufficient to explain the pattern of age-related variation in sex ratio we observed in our study population.

Variation in TSR of different cohorts may occur due to sex-related impact of temporally varying ecological conditions, and considerable inter-annual variation (ca. 9%) in yearlings TSR was in fact observed in our study. We used adult survival as a proxy to ecological conditions encountered by our population year-round to investigate the sex-related effects of adverse conditions. Reduction in survival was accompanied by increased male-bias among the yearlings. Hence, before sexual maturation females incur larger ‘base-line’ mortality but such viability gap widens under the conditions that cause increased mortality. Because monitoring survival of barn swallows during migration and wintering has not yet been possible, the stage in the annual cycle when differential mortality occurs is unknown. Larger female mortality under adverse conditions may represent the sex-dependent expression of carry-over effects of asymmetries in competition between nestlings of either sex: females are known to be competitively inferior to their male siblings [41] and the consequence of such asymmetry may become manifest under harsh post-fledging conditions during migration or wintering [34]. Alternatively, larger survival costs may arise during migration owing to sex-related differences in morphology (e.g. wing loading) [42]. No sex-related variation in timing of molt has been observed in the main wintering and molting area, centered in western equatorial Africa, of barn swallows that breed in Italy [40], suggesting that sex-related mortality does not result from divergence in molt schedule. Yet, retaining the same molt schedule may entail either sex with different costs, again causing variable sex-related mortality depending on conditions during wintering. On the other hand, we could not identify any obvious process that could have resulted in a spurious association between yearling TSR and survival. For example, it is not obvious how variation in secondary sex ratio could result in a negative covariation between parental survival and TSR of yearlings given that adult survival is not predicted by offspring TSR at the population level (our unpublished data; also see [20]).

The negative association between survival and TSR was consistent with that expected based on most studies of small or declining bird populations, where an (increasing) excess of males has been documented [3]. However, several of the mechanisms that have been invoked to explain this pattern (e.g. sex-related effects of inbreeding, social dominance or predation) seem not to apply to our model population, given its large genetic size, no social hierarchy in access to food and no evidence of sex-related predation (see also above). Hence, the association between negative population trends and TSR may occur under a wider spectrum of conditions than is commonly thought [3].

Severe decline has been recorded in the size of the populations of several bird species breeding in Europe, and such negative population trends have been shown to be particularly pronounced for long-distance migratory species that breed in farmland habitats, like the barn swallow we studied [43]–[48]. Whenever possible, monitoring programs of breeding bird populations should therefore also investigate temporal variation in TSR. Yet, it should be noted that whether change in TSR is a cause or, conversely, an effect of population trends is largely unknown (see Introduction).

In species with natal and/or breeding dispersal, spatial variation in ecological conditions (e.g. habitat quality, population density and sex ratio) can have a major impact on successful reproduction because decisions on where to settle can affect availability of limiting resources or mates (e.g. [49]). In the barn swallow, appropriate colony choice by male yearlings can affect mating opportunities, whereas colony choice by females will have markedly smaller consequences for their mating opportunities owing to generalized male-bias in the population. Because older individuals arrive earlier to their breeding colony, yearling males can have a clue to the extent of male-bias in local TSR, and thus of local mating and sperm competition by visiting potential breeding sites. Obviously, choice of last arriving yearlings will be affected by the sex ratio of older individuals as well as by that of yearlings that have already settled. Detailed information on individual arrival date is difficult to acquire and was not available for all the individuals in the sample. Yet, we are convinced that TSR of older individuals is a reliable proxy for local TSR experienced by yearlings because influx of new-comers should preserve the initial ranking of TSR among colonies while gradually reducing their reciprocal differences in TSR, thus making our approach conservative. Moreover, it must be emphasized that a negative covariation between contemporary sex ratios of yearlings and older individuals was consistently observed in the large majority (13/14) of the study colonies.

The negative association between TSR of new-settler yearlings and TSR of older individuals thus supports the ‘arrival TSR’ hypothesis (see Introduction) and strongly suggests that local sex ratio is a major factor affecting dispersal decisions of yearlings, with potential cascading effects on local sex ratio in the following years, owing to very high breeding philopatry. Hence, local TSR not only can drive decisions on whether or not to disperse from the natal site [50] but also decisions on where to settle. Conversely, we could find no support for the ‘departure TSR’ hypothesis that offspring choose their breeding colony by exploring different sites soon after fledging, before their first migration to Africa.

A great deal of attention has been devoted to the ecological and evolutionary determinants of primary and secondary sex ratio variation at the family level (e.g. [51]), while insights into the extent and mechanisms that govern variation in TSR at population and local scale has been lagging behind. In this study, we contributed to filling this gap by showing that, as in most bird populations, barn swallows have male-biased TSR but this is caused by larger female mortality before rather than after sexual maturation. Male bias among yearlings was more pronounced after large population declines, suggesting that males are less susceptible to the ecological effects that cause negative population trends. This implies that deterioration of ecological conditions can predictably lead to an increase in male bias in TSR among yearlings, with consequences for sexual selection processes and population trends. At the local scale our study also disclosed a mechanism that governs colony TSR, which consists of the effect that sex ratio of philopatric, relatively old breeders has on decisions of where to settle by dispersing yearlings. Thus, in spatially structured populations natal dispersal can homogenize local TSR because individuals tend to avoid settling in places where individuals of their sex are over-represented.
